# Liposomal formulations of anticancer copper(ii) thiosemicarbazone complexes†

**DOI:** 10.1039/d1dt02763h

**Published:** 2021-10-07

**Authors:** Marlene Mathuber, Sonja Hager, Bernhard K. Keppler, Petra Heffeter, Christian R. Kowol

**Affiliations:** Institute of Inorganic Chemistry, Faculty of Chemistry, University of Vienna Waehringer Straße 42 1090 Vienna Austria christian.kowol@univie.ac.at; Institute of Cancer Research and Comprehensive Cancer Center, Medical University of Vienna Borschkegasse 8A 1090 Vienna Austria; Research Cluster “Translational Cancer Therapy Research”, University of Vienna and Medical University of Vienna 1090 Vienna Austria

## Abstract

α-N-Heterocyclic thiosemicarbazones such as triapine and COTI-2 are currently investigated as anticancer therapeutics in clinical trials. However, triapine was widely inactive against solid tumor types. A likely explanation is the short plasma half-life time and fast metabolism. One promising approach to overcome these drawbacks is the encapsulation of the drug into nanoparticles (passive drug-targeting). In a previous work we showed that it was not possible to stably encapsulate free triapine into liposomes. Hence, in this manuscript we present the successful preparation of liposomal formulations of the copper(ii) complexes of triapine and COTI-2. To this end, various drug-loading strategies were examined and the resulting liposomes were physico-chemically characterized. Especially for liposomal Cu–triapine, a decent encapsulation efficacy and a slow drug release behavior could be observed. In contrast, for COTI-2 and its copper(ii) complex no stable loading could be achieved. Subsequent *in vitro* studies in different cell lines with liposomal Cu–triapine showed the expected strongly reduced cytotoxicity and DNA damage induction. Also *in vivo* distinctly higher copper plasma levels and a continuous release could be observed for the liposomal formulation compared to free Cu–triapine. Taken together, the here presented nanoformulation of Cu–triapine is an important step further to increase the plasma half-life time and tumor targeting properties of anticancer thiosemicarbazones.

## Abstract 2

Liposomal formulations of copper(ii) complexes of α-N-heterocyclic thiosemicarbazones (triapine and COTI-2) were synthesized, characterized and biologically investigated, with the aim to enhance their plasma half-life time and tumor accumulation.

## Graphical abstract

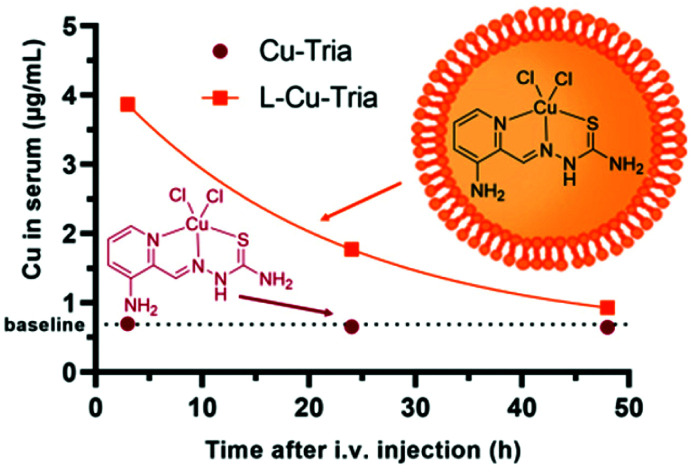

## Introduction

**Fig. 1:**
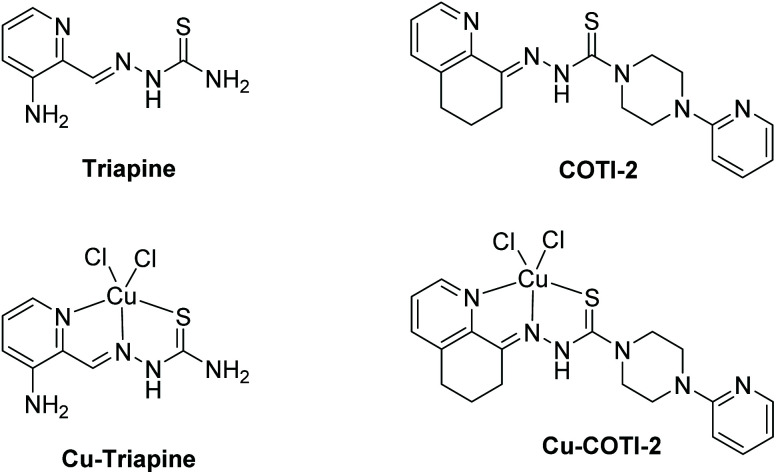
Chemical structures of the clinically investigated thiosemicarbazones triapine and COTI-2 as well as their copper(ii) complexes.

α-N-Heterocyclic thiosemicarbazones possess a distinctive N,N,S-donor ligand set, which characterizes them as strong metal chelators.^1^ Both, the free ligands as well as their metal complexes (*e.g.* with Cu, Fe, Ru, Ga) exhibit extraordinary antibacterial, antiviral and antitumor activity.^2^ Regarding their mode of action as anticancer drugs, recent studies proposed that thiosemicarbazones can impact on several iron-dependent biological pathways by chelation of both iron(ii) and iron(iii) ions. Thus, they are generally considered as “iron-interacting” drugs.^3,4^ Moreover, another important mechanism (for at least a part) of this compound class is their interaction with cellular copper ions.^5–7^ 3-Aminopyridine-2-carboxaldehyde thiosemicarbazone (triapine; Fig. 1) is the most prominent and well-studied representative, with its main target being the iron-dependent enzyme ribonucleotide reductase (RR).^3,4^ Triapine has been already evaluated in more than 30 clinical phase I/II trials against different cancer types.^8^ Noteworthy, recently triapine entered a clinical phase III study in combination with cisplatin and radiation therapy against cervical or vaginal cancer (study number NCT02466971, clinicaltrials.gov).^9^ Although triapine showed especially promising results against hematological cancers (*e.g.* advanced leukemia)^10^ hardly any activity was found against solid cancers (*e.g.* renal cell carcinoma or non-small-cell lung cancer).^11,12^ The inefficacy of triapine most likely results from rapid metabolism/excretion^13^ and/or insufficient tumor accumulation.^14,15^ More recently, also new thiosemicarbazones have been clinically investigated, namely di-2-pyridylketone 4-cyclohexyl-4-methyl-3-thiosemicarbazone (DpC) and 4-(pyridine-2-yl)-*N*-([(8*E*)-5,6,7,8-tetrahydroquinolin-8-ylidene] amino)piperazine-1-carbothio-amide (COTI-2; Fig. 1). DpC entered a phase I clinical trial for patients with advanced solid tumors in 2016 (study number NCT02688101, clinicaltrials.gov).^16^ COTI-2 is currently studied in a phase Ib/IIa clinical trial for the treatment of gynecologic malignancies (study number NCT02433626, clinicaltrials.gov).^17^ COTI-2 showed nanomolar cytotoxicity against various cancer cells *in vitro* and promising activity *in vivo*.^18^ Biological investigations revealed that COTI-2 could restore the tumor suppressor functionality of mutated p53.^19,20^

A general approach to distinctly prolong the plasma half-life time and simultaneously increase the tumor-specificity/accumulation (also resulting in reduced adverse effects), is the encapsulation of therapeutics into nanoparticulate drug formulations such as liposomes.^21^*Via* the enhanced permeability and retention (EPR) effect nanoformulations can accumulate in tumor tissue (by passive targeting) due to the combination of leaky blood vessels together with a defective lymphatic drainage system.^22,23^ Several liposomal formulations of anticancer drugs are already approved, such as Doxil© (liposomal doxorubicin), Depocyt© (liposomal cytarabine) and Marqibo© (liposomal vincristine).^24^ Previously, we already synthesized the first liposomal nanoformulations of triapine. However, a burst release of the drug occurred and no stable encapsulation could be achieved.^21^

In this work, we investigated, if the copper(ii) complex of triapine (Cu–Tria; Fig. 1) is a suitable derivative for encapsulation into liposomes. The underlying idea is that Cu–Tria can act as a “prodrug” with release of the triapine ligand after reduction.^5,25^ Furthermore, we investigated the encapsulation potential of COTI-2 and its copper(ii) complex (Cu–COTI; Fig. 1). Drug loading was performed either by the remote loading approach (also used for preparation of *e.g.* the clinically approved Doxil©^26^) or by adding the drugs directly to the lipid mixture.^27^ The obtained liposomal formulations were physico-chemically characterized and subsequently tested for their anticancer activity on different cancer cell lines in comparison to the free ligands and their copper complexes. In addition, the impact on methemoglobin formation (a common side effect of triapine in clinical trials) was examined.^28^ Finally, the plasma levels *in vivo* have been analyzed to prove an enhanced retention time of the liposomal drugs compared to the free complexes.

## Results and discussion

### Liposome preparation

**Scheme 1:**
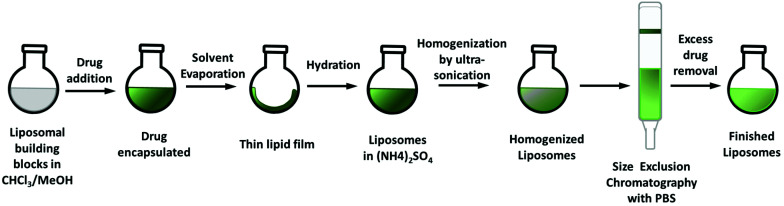
Preparation of the liposomal formulations by addition of the drug at the beginning of the synthetic procedure.

**Scheme 2:**
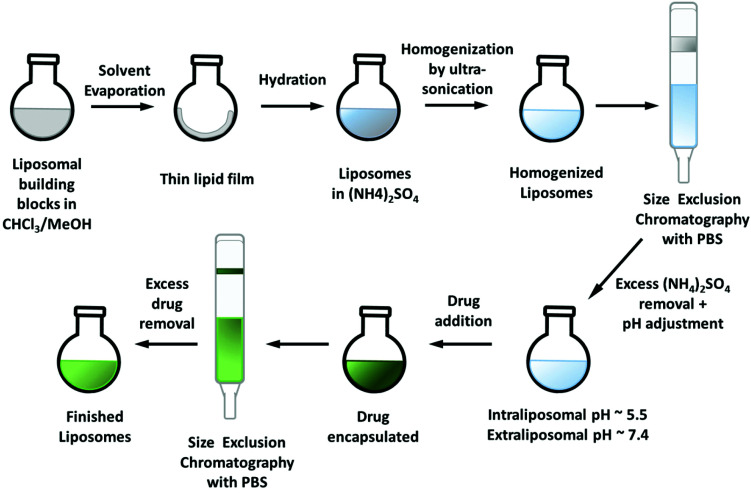
Preparation of the liposomal formulations by the remote-loading approach.

The liposomal preparation method is crucial for the final properties of the nanoparticles such as particle size and encapsulation efficacy (EE). In this work, two methods were applied: (1) The respective drug is added to the lipid mixture at the beginning of the preparation (Scheme 1), commonly applied for highly lipophilic (and water-insoluble) compounds.^27^ (2) The remote-loading approach which is preferably used for (fairly) water-soluble compounds (Scheme 2).^29^ For both methods the liposomal building blocks DSPC/CHOL/DSPE–mPEG (2000) 55 : 40 : 5 mol mol^−1^ (DSPC = 1,2-distearoyl-*sn-glycero*-3-phosphocholine; CHOL = cholesterol; DSPE–mPEG 2000 = 1,2-distearoyl-*sn-glycero*-3-phosphoethanolamine-*N*-[methoxy(polyethylene glycol)-2000)] were used, a formulation, which is well established in literature.^21,26,30^

COTI-2 is highly lipophilic (log *P* of +2.89)^31^ and therefore suitable for the approach depicted in Scheme 1. Briefly, the building blocks and the drug were dissolved together in a mixture of chloroform and methanol. After refluxing at 65 °C for 1.5 h, the solvents were removed and the thin film of lipids was dried *in vacuo*. This film was then rehydrated in an aqueous solution of choice (*e.g.* 0.3 M (NH_4_)_2_SO_4_) to form the liposomes (so called thin lipid hydration method).^32^ To achieve the required size reduction and homogenization, subsequently ultra-sonication with a micro-tip was performed. Non-encapsulated drug was removed from the liposomal formulation by size exclusion chromatography (SEC) using the Sephadex G50 dextran matrix and phosphate-buffered saline (PBS) at pH 7.4. Hardly any free COTI-2 could be observed on the column correlating with a high encapsulation efficacy. However, overnight (despite storage at 4 °C) large amounts of precipitate formed, indicating a burst release of COTI-2. A switch in the rehydrating solvent from (NH_4_)_2_SO_4_ to KCl (0.15 M) did not change the encapsulation stability. Therefore, we next tried to encapsulate its copper(ii) complex, which was synthesized from a 1 : 1 mixture of COTI-2 and CuCl_2_ in methanol^31^ bearing a neutral thiosemicarbazone and two chlorido ligands (Fig. 1). However, similar results to the free COTI-2 were obtained, a high EE followed by rapid precipitation, which could not even be prevented by storage at −20 °C. Therefore, we decided to change the metal salt by *in situ* complexation of COTI-2 with either Cu(NO_3_)_2_ or CuSO_4_. Subsequently, the liposomal building blocks were added to the respective reaction solution and synthesis continued as described above. By complexation with Cu(NO_3_)_2_, no stable encapsulation could be obtained either. However, applying the same approach to the complex synthesized with CuSO_4_, we succeeded in a stable liposomal formulation (named l-Cu–COTI; Fig. S1†), which was further characterized and biologically tested (see below). Unfortunately, only a low EE of 21 ± 3% (*n* = 5) was achieved for l-Cu–COTI. No precipitations could be observed up to two weeks when stored at 4 °C.

After successful encapsulation of Cu–COTI, we tried the same approach also for the synthesis of liposomal formulations of Cu–Tria (as mentioned before, it was not possible to stably encapsulate free triapine into liposomes^21^). To this end, we used the preformed Cu(triapine)Cl_2_ complex as well as the *in situ* complexation of triapine with CuSO_4_. Nonetheless, no sufficient encapsulation of the complexes could be observed, probably due to a distinctly higher hydrophilicity compared to Cu–COTI. We also experimented with complexation directly in the liposomes by adding triapine to a liposomal formulation with an intra-liposomal copper solution. However, this resulted in complex formation mainly outside the liposomal particles. Therefore, we changed the general method to the remote-loading approach (Scheme 2).^29^

*Via* an ammonium sulfate gradient (lower intra-liposomal pH compared to the pH of the extra-liposomal solution) the drug can be “absorbed” into the liposomes.^33^ The drugs should have a log *D*_7.0_ in the range of −2.5 to 2 and a p*K*_a_ ≤ 11, to be suitable for this technique.^29^ For the remote-loading approach, the thin lipid film was prepared as described before,^27^ however, without addition of the drug. Rehydration was again performed with a 0.3 M (NH_4_)_2_SO_4_ solution and size reduction by ultra-sonication. Afterwards, size exclusion chromatography (Sephadex G50 Fine) with PBS at pH 7.4 was carried out to remove any access of (NH_4_)_2_SO_4_ and create the required pH gradient (intraliposomal pH ∼5.5, extraliposomal pH ∼7.4). Cu–Tria (synthesized from a 1 : 1 mixture of triapine and CuCl_2_ ^34^) was encapsulated by addition to the freshly prepared liposomes and the resulting solution was stirred for 1 h at 65 °C. A second SEC column (same conditions as before) removed unloaded drug and generated the liposomal formulation of Cu–Tria (Fig. S1†). Indeed, hardly any free drug retained on the column and the formed liposomes were stable. Despite the successful encapsulation, the EE varied over a series of experiments. Since the performance of the remote-loading approach strongly depends on the right pH gradient, we analyzed the pH value after addition of Cu–Tria to the liposomal solution and a pH drop from 7.2 to ∼6.9 could be observed. This could be circumvented by using higher volumes of the liposomal solution (3.5 mL), maintaining the essential intra-liposomal (∼pH 5.5) *versus* extra-liposomal (∼pH 7.2) pH difference. Additional modifications such as increasing the buffer strength or re-buffering the liposomal solution after addition of Cu–Tria did not result in higher EE, even when increasing the amount of drug. Consequently, to reach a higher drug loading, the volume of the solution was reduced to ∼1/2 on a rotary evaporator. Afterwards, another SEC column separation was carried out to ensure that Cu–Tria was still completely encapsulated. No free drug could be observed on this column, indicating a stable encapsulation and no drug release during this step. With this modification in the protocol, an EE of 64 ± 5% (*n* = 15) with high reproducibility and high stability (no precipitate formed after >70 days at 4 °C) could be obtained.

The improved encapsulation properties of Cu–Tria (compared to free triapine) is assumed to be based on the formation of positively charged complexes after release of the chlorido ligand(s) and coordination of a water molecule.^35^ In this case the neutral chlorido complex would enter the liposomes (remote loading approach), where it is hydrolyzed with formation of [CuL]^+^. Also in case of COTI-2, the *in situ* preformed copper complex using CuSO_4_ was the best candidate for liposomal loading (using the direct encapsulation method). The fast precipitation of free COTI-2 after successful encapsulation can probably be explained by the very low aqueous solubility. Even when traces are released from the liposomes, they will precipitate in the environmental aqueous solution building a driving force toward more and more compound release.

### Liposome characterization

**Fig. 2:**
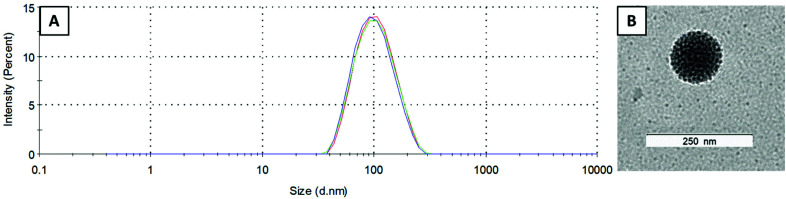
(A) Size distribution of l-Cu–Tria (by intensity) measured by DLS (each line represents measurements in triplicate). (B) Transmission electron microscopy (TEM) image of l-Cu–Tria; samples were prepared by negative staining with Uranyless.

One of the most important characteristics of liposomes is their size and size distribution, which can be examined by dynamic light scattering (DLS). Measurements were performed in PBS (pH 7.4) at room temperature and the average size as well as polydispersity index (PDI) were highly reproducible (in general, a PDI below 0.15 indicates a narrow size distribution). The Cu–COTI-loaded liposomes showed a size of 99 ± 1 nm and a PDI of 0.09 ± 0.01 (Fig. S2†). For the liposomes with encapsulated Cu–Tria, a size of 90 ± 2 nm and a PDI of 0.12 ± 0.02 were obtained (Fig. 2A). After reduction of the volume, we again checked the size and size distribution of l-Cu–Tria by DLS to confirm that there was no degradation or agglomeration of the liposomes (Fig. S3†). The average size and PDI of l-Cu–Tria was well reproducible with a size of 93 ± 5 nm and a PDI of 0.13 ± 0.02 over the synthesis of 15 batches.

In addition, the zeta potential (= the electrostatic repulsion of the particle surface) was examined in PBS (pH 7.4), resulting in slightly negative values for l-Cu–Tria at −1.3 ± 0.6 mV and for l-Cu–COTI at −1.5 ± 0.8 mV, which can be expected for PEGylated liposomes.^36,37^

Furthermore, we examined the morphology of l-Cu–Tria by negative stain transmission electron microscopy (TEM) measurements. A representative picture can be seen in Fig. 2B (the whole image can be found in Fig. S4†), which nicely depicts the spherical shape of the liposomes and confirms the size of ∼100 nm measured by DLS.

### Drug release from liposomes

To investigate the drug release of l-Cu–Tria, the dialysis diffusion technique was applied. To this end, the liposomal formulation was transferred into a dialysis bag (molecular weight cut-off 14 kDa), which was then immersed into a PBS solution (pH 7.4) at 37 °C. Over a period of 48 h, samples were removed from this solution and the released drug amount was measured by UV-Vis spectroscopy. In addition, reference measurements with Cu–Tria only were performed under the same conditions. In these experiments a low release of 9% after 48 h was observed, which indicates stable drug encapsulation and a controlled release behavior over time. In contrast, free Cu–Tria showed the expected fast release out of the dialysis bag of nearly 100% already after 1 h. This method could not be applied for the evaluation of l-Cu–COTI, since the free Cu–COTI complex possesses hardly any aqueous solubility and immediately precipitates after release. Therefore, the stability of the l-Cu–COTI encapsulation was indirectly evaluated *via* their biological activity on different cancer cell lines.

### Biological investigations

#### Anticancer activity and uptake of l-Cu–Tria in human cancer cells

**Fig. 3:**
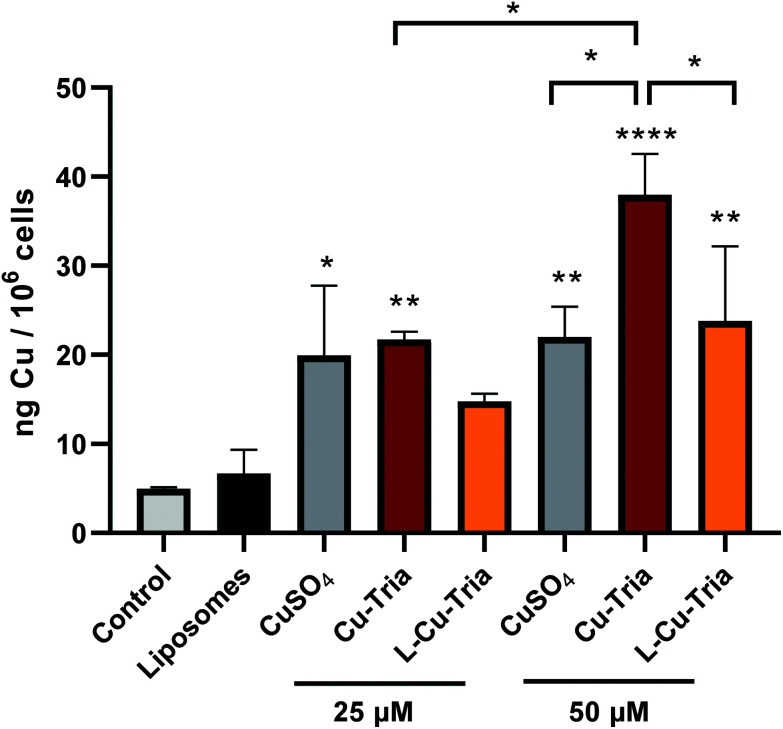
Cellular copper levels of SW480 cells measured by ICP-MS after treatment with empty liposomes, CuSO_4_, triapine, Cu–Tria or l-Cu–Tria at the indicated concentrations for 3 h. Values given are the mean ± standard deviation (SD) of triplicates. Significance to control (stars above bars) and to other treatments (stars above brackets between bars) was calculated by one-way ANOVA and Tukey's multiple comparison test (* *p* < 0.05; ** *p* < 0.01; **** *p* < 0.0001).

It is well known from the literature,^38–40^ that a PEGylated surface reduces the attachment of liposomes to the plasma membrane and, consequently, hampers endocytic cellular uptake, *e.g.*, *via* macrophages. Thus, such nanocarriers are frequently referred to as “stealth liposomes”. Noteworthy, this type of liposomes is also characterized by a distinctly reduced uptake into cancer cells. In fact, their *in vivo* mode of action seems to be based mainly on enhanced accumulation in the malignant tissue by the EPR effect followed by continuous drug release over a longer time.^38,40^ To evaluate whether our thiosemicarbazone nanoformulations act as “stealth liposomes”, ICP-MS measurements of intracellular copper levels of cancer cells treated for 3 h with either the free copper complex or the liposomal formulation were performed. Indeed, at 50 μM significantly lower copper level were detected in l-Cu–Tria-treated cells compared to Cu–Tria (Fig. 3), confirming the “stealth nature” of the nanocarriers.

**Fig. 4:**
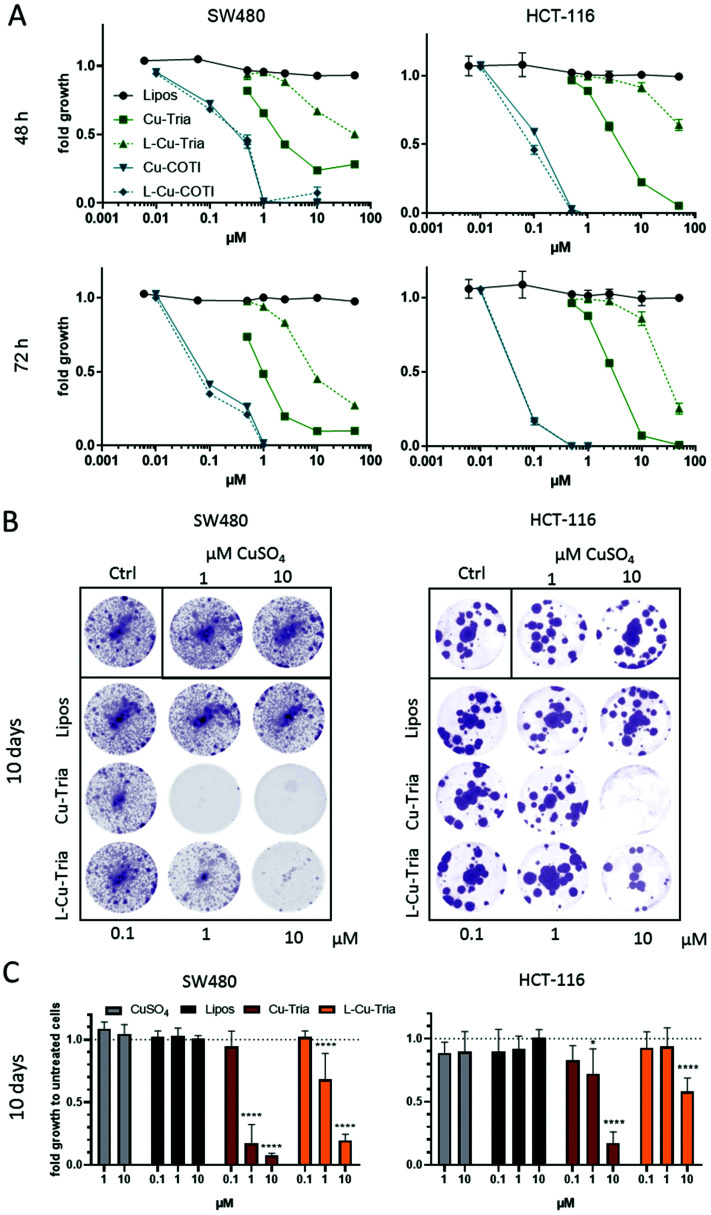
(A) Cell viability of SW480 and HCT-116 cells treated with free complexes, drug-free or -loaded liposomes measured by MTT assay after 48 and 72 h. Values given in the graph are the mean ± SD of triplicates from one representative experiment out of three. (B) Cell viability after long-term treatment of SW480 and HCT-116 cells with CuSO_4_, free complexes, drug-free or -loaded liposomes. After 10 days, cells were fixed and stained with crystal violet. Representative images of stained cells (violet) are shown. Fold growth to untreated cells was calculated from integrated density analyzed with Image J and shown in (C). Values given are the mean ± SD of duplicates from three experiment. Significance to control was calculated in with two-way ANOVA and Dunnett's multiple comparison test (**** *p* < 0.0001, * *p* ≤ 0.05).

The biological activity of the liposomal formulations compared to the free complexes was studied by MTT viability assays in SW480 and HCT-116 cells after 48 and 72 h drug treatment (Table 1 and Fig. 4A). In comparison, also the activity of the metal-free ligands (triapine and COTI-2) as well as the unloaded liposomes was analyzed (Table 1 and Fig. 4A). In general, unloaded liposomes had no observable effects on cell viability. While for triapine complexation with copper decreased its anticancer activity in both cell models and time points, for COTI-2, the copper complex displayed similar or even increased activity (Table 1), which is in line with previous publications.^5,31^l-Cu–Tria displayed an over 7- and 14-fold lower anticancer activity (in HCT-116 and SW480 cells, respectively) compared to free Cu–Tria after 48 h of treatment. This is in agreement with the lower drug uptake (Fig. 3), but additionally reveals that the complexes are stably encapsulated into the liposomes, protecting the cells from the cytotoxic activity. After 72 h, the difference decreased especially in SW480 cells to an 8-fold lower anticancer activity, indicating drug release from the liposomes over several days. Further prolongation of the exposure time to 10 days additionally enhanced the activity of l-Cu–Tria (Fig. 4B and C).

**Table 1:** IC_50_ values (mean ± SD) after 48 and 72 h of the metal-free ligands, the free complexes, CuSO_4_ and their liposomal formulations in SW480 and HCT-116 cells

	SW480 IC_50_ (μM)		HCT-116 IC_50_ (μM)	
	48 h	72 h	48 h	72 h
Liposomes	>50	>50	>50	>50
CuSO_4_	>50	>50	>50	>50
Triapine	0.91 ± 0.08	0.77 ± 0.10	1.40 ± 0.69	0.81 ± 0.07
Cu–Tria	2.99 ± 1.66	0.90 ± 0.14	5.53 ± 0.57	3.38 ± 0.45
l-Cu–Tria	>44	7.29 ± 1.93	>42	26.77 ± 8.10
Ratio l-Cu–Tria/Cu–Tria	>14.7	8.0 ± 1.2**	>7.6	7.9 ± 1.9*
COTI-2	n.d.	0.30 ± 0.06	n.d.	0.06 ± 0.02
Cu–COTI	0.44 ± 0.06	0.09 ± 0.01	0.13 ± 0.05	0.07 ± 0.01
l-Cu–COTI	0.43 ± 0.06	0.12 ± 0.06	0.09 ± 0.03	0.06 ± 0.02
Ratio l-Cu–COTI/Cu–COTI	0.9 ± 0.2^n.s^	0.8 ± 0.2^n.s^	0.7 ± 0.2^n.s^	0.8 ± 0.2^n.s^

With regards to the liposomal COTI formulation, l-Cu–COTI, cell viability was similar when compared to the treatment with free Cu–COTI (Table 1). This indicates a fast release of the drug from the liposomes and, consequently, a rather low loading stability. Consequently, l-Cu–COTI has not the appropriate drug release profile that allows a stable transport of the intact liposomes into the malignant tissue, where it is released in a tumor-specific manner. Based on these results, no further *in vitro* or *in vivo* experiments were performed with this drug.

**Fig. 5:**
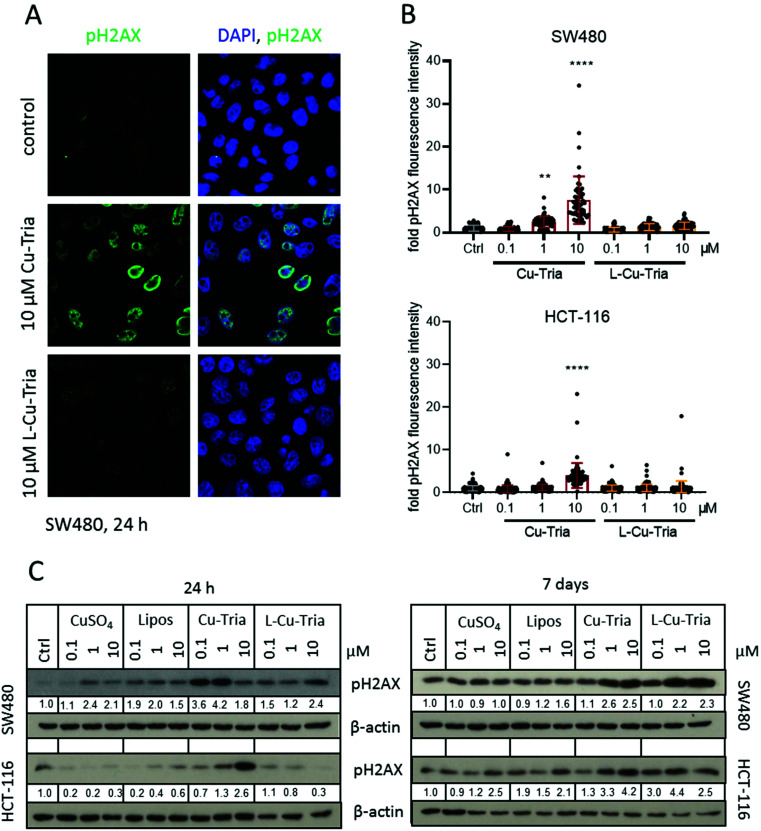
(A) Representative images of pH2AX immunofluorescence staining (green) in nuclei (DAPI, blue) of SW480 cells treated as indicated. (B) Quantification of immunofluorescence intensities in the nucleus of the DNA damage marker pH2AX in SW480 and HCT-116 cells treated with the indicated drugs and concentrations for 24 h. Values given are the mean fold intensities ± SD per nucleus. Significance to control was calculated with two-way ANOVA and Dunnett's multiple comparison test (**** *p* < 0.0001, ** *p* ≤ 0.01). (C) Western blot analysis of pH2AX expressed by SW480 and HCT-116 cells treated with indicated drugs and concentrations 24 h and 7 days. β-Actin was used as a loading control. Normalized pH2AX values to β-actin and compared to control are given below the bands.

The prolonged replication stress induced by the RR inhibition of triapine was previously reported to result in DNA double-strand breaks, assumingly due to the collapse of DNA replication forks.^41^ Accordingly, also when looking at pH2AX (by immunofluorescence staining and western blot analysis) as a marker for DNA damage and triapine activity, encapsulation into liposomes prevented DNA damage generated by triapine-induced RR inhibition in 24 h experiments (Fig. 5). CuSO_4_ and the empty liposomes had either no or only minor effects in these experiments (Fig. 5C and Fig. S5†). However, upon prolonged incubation (7 days), the pH2AX levels became similar (HCT-116 cells) or even higher (SW480 cells) in the l-Cu–Tria samples compared to the free copper complex (Fig. 5C). This indicates a slow but continuous release of Cu–Tria from the liposomes.

#### Impact of the liposomal formulation on the serum half-life time *in vivo*

**Fig. 6:**
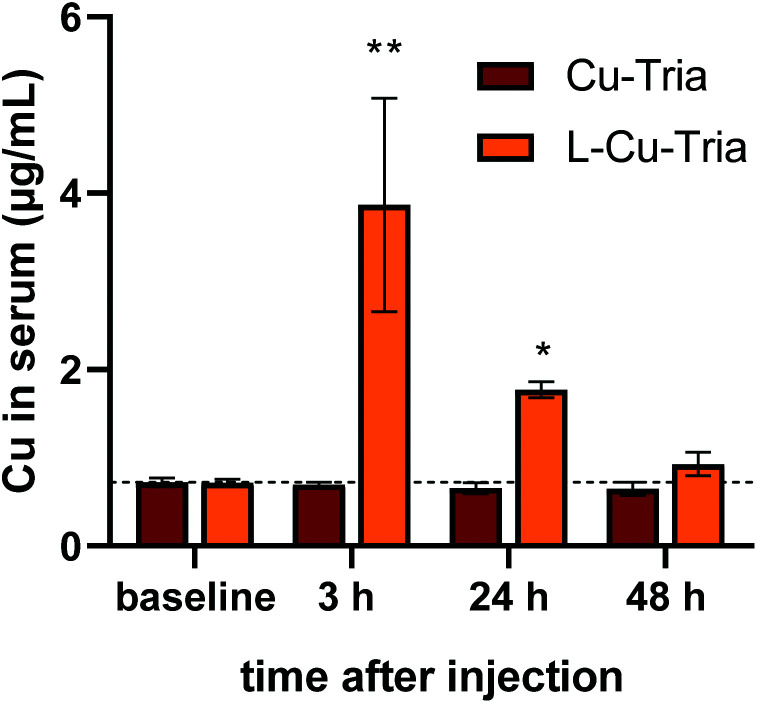
Serum copper levels of female Balb/c mice treated i.v either with 1.75 mg kg^−1^ Cu–Tria or l-Cu–Tria and blood drawn *via* the facial vein at the indicated time points. Samples for baseline were collected from the same mice 7 days prior drug treatment. Values given are mean ± standard error of the mean (SEM) and significance was calculated by Kruskal–Wallis test against the baseline with false discovery rate by Benjamini and Hochberg (* *p* < 0.05; ** *p* < 0.01).

To get first indications on the pharmacokinetics of l-Cu–Tria in comparison to Cu–Tria, copper levels in serum of mice treated with equimolar drug concentrations were determined *via* ICP-MS. As a first step, the basal serum levels of animals (0.7 μg Cu mL^−1^ serum) were assessed 7 days before treatment. Then the mice were treated intravenously (i.v.) with 1.75 mg kg^−1^ Cu–Tria either as a free complex or in form of the liposomal formulation and blood was collected by the facial vein after 3, 24 and 48 h (Fig. 6). Treatment with Cu–Tria did not result in measurable changes of the serum copper levels at the investigated time points. In contrast, injection of the liposomal Cu–Tria increased serum copper levels significantly. Thus, after 3 h a serum copper concentration of around 4 μg mL^−1^ was detected, which decreased to 1.7 μg mL^−1^ after 24 h and returned to baseline copper levels after 48 h (1 μg mL^−1^). In general, the undetectable copper levels upon treatment with the free complex were not unexpected, as we (and others) previously reported that the serum half-life time of metal-free triapine is below 1 h in mice based on the relatively fast excretion/metabolism of the drug.^13,14^ In addition, there is already a rather high copper content in the serum (due to copper-containing enzymes such as ceruloplasmin and albumin),^42,43^ which decreases the sensitivity of the measurements. However, considering that the theoretical elevation of copper blood levels by the drugs at time of injection would be ∼5 μg mL^−1^, our preliminary data indicate that l-Cu–Tria is characterized by a distinctly prolonged serum half-life (>10 h). This would be in good agreement with other liposomal drugs such as DOXIL®, which had a reported serum half-life of 13 h in female C57BL/6 mice.^44^

#### Investigation of methemoglobin formation

**Fig. 7:**
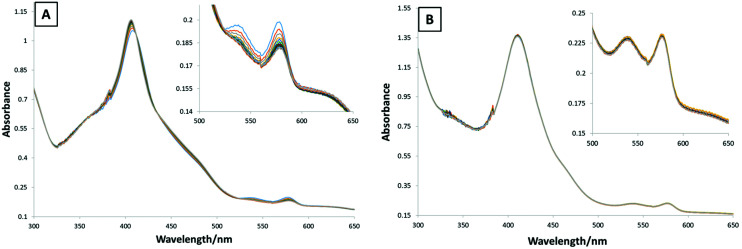
Time resolved UV-Vis spectroscopy of the reaction between human HbO_2_ and (A) triapine and (B) l-Cu–Tria in the presence of iron(iii) during 21 min (inset zoom of the range 500–650 nm).

The occurrence of methemoglobinemia, where oxyhemoglobin (HbO_2_) is oxidized to methemoglobin (metHb), is a side effect frequently observed in clinical trials of triapine.^45^ This is caused by the iron(iii) triapine complex, which is formed in the bloodstream and, subsequently, can oxidize hemoglobin.^46^ We investigated, therefore, whether l-Cu–Tria in the presence of iron(iii) leads to the formation of metHb. To this end, l-Cu–Tria and triapine, which was included as positive control, were incubated with Fe(NO_3_)_3_ in a 2 : 1 ration for 10 min at r.t. The subsequent reaction with HbO_2_ was monitored by UV-Vis spectroscopy.^47,48^ For triapine the typical decrease in the absorption of the HbO_2_ bands (at 539 and 577 nm) and the hypsochromic shift could be observed (Fig. 7A), which indicates the formation of metHb and is comparable with the results of our previous work.^21^ For l-Cu–Tria no changes in the UV-Vis spectra were measured over time, suggesting that no redox-active iron(iii) complex was formed, in agreement with the high stability constant of Cu–Tria^35^ (also in case of Cu–Tria alone no redox reaction occurred, data not shown). Therefore, this data demonstrates the stable complexation of triapine in form of Cu–Tria preventing the generation of methemoglobinemia. Noteworthy, COTI-2 and Cu–COTI could not be investigated due to their very low aqueous solubility. However, for COTI-2 methemoglobinemia has not been listed as side effect in the first preliminary report of the clinical phase I trial.^49^ This clinical observation is in line with the work of Basha *et al*., which proposed that thiosemicarbazones with a substituted terminal –NH_2_ group have a diminished interaction with haemoglobin and, thus, are not likely to cause methemoglobinemia.^46^

## Conclusions

α-N-Heterocyclic thiosemicarbazones are promising anticancer drugs and various compounds have already been studied in clinical trials. Nevertheless, the most prominent representative triapine did not exhibit distinct antitumor activity against most solid tumor types. The underlying reasons are most probably rapid metabolization and a short plasma half-life time. To increase the retention of a drug in the body and simultaneously enhance its concentration at the tumor site, the passive drug targeting approach represents a promising and frequently used strategy. The drug is encapsulated into nanoparticles, which then accumulate in the tumor tissue utilizing the EPR effect. This concept is already successfully applied in the clinics *e.g.*, for doxorubicin, daunorubicin, vincristine and irinotecan.^24^ For the development of nanocarriers, several parameters such as size, zeta potential, EE and reproducibility have to be considered.^50^ Most importantly, the drug release profile, which is crucial for the therapeutic effectiveness, has to be balanced: on the one hand, the drug is inactivated by encapsulation into the nanoformulation, on the other hand, a too rapid release leads to loss of selectivity. We demonstrated in a previous study that free triapine is not suitable for this method, as a fast drug release from the nanoformulations could be observed.^21^ Therefore, in this work we chose the copper(ii) complex (Cu–Tria) as derivative for encapsulation, since it can be considered as prodrug, releasing triapine after reduction to copper(i).^5,25^ Additionally, the clinically studied third-generation thiosemicarbazone COTI-2 and its copper(ii) complex (Cu–COTI) were investigated. Owing to the lipophilic nature of COTI-2, the most suitable technique for encapsulation was the addition of the drug to the thin lipid film at the beginning of the synthesis. As for the more water-soluble Cu–Tria, the remote-loading approach (used for the preparation of Doxil©) was applied. *In vitro* tests confirmed the stable encapsulation and slow drug release behavior of l-Cu–Tria, in line with DNA damage experiments. However, for the liposomal formulations of Cu–COTI no difference in the *in vitro* activity to the free drugs could be observed, indicating a fast release from the nanocarrier. This clearly shows that the exact chemical structure and properties strongly impacts on the ability to generate stable liposomal formulations (Cu–Tria *vs.* Cu–COTI and Cu–Tria *vs.* triapine). Consequently, *in vivo* tests were only performed with l-Cu–Tria and indeed a distinctly prolonged half-life time compared to Cu–Tria was achieved. Furthermore, the formation of methemoglobin, which is a side effect from triapine, could be completely prevented when using l-Cu–Tria. Taken together, we could show that liposomal formulations are a promising method to increase the plasma half-life time of Cu–Tria, which is an important step further to successfully generate thiosemicarbazone nano-preparations.

## Materials and methods

### Chemicals

All solvents and reagents were purchased from commercial suppliers. They were, unless stated otherwise, of analytical grade and used without further purification. 1,2-Distearoyl-*sn-glycero*-3-phosphocholine (DSPC), 1,2-distearoyl-*sn-glycero*-3-phosphoethanolamine-N-[methoxy(polyethylene glycol)-2000] (ammonium salt) (DSPE–mPEG(2000)) and cholesterol were obtained from Avanti Polar Lipids Inc. (Alabaster, AL). The dextran matrix Sephadex G-50 Fine was used for the separation of the loaded liposomes from the free drug and to generate the required pH gradient. Its fractionation range lies between a molecular mass of 1500 to 30 000 Da and is, therefore, ideal for liposomal formulations. Human hemoglobin was acquired from Sigma Aldrich. Milli-Q water was obtained from a Millipore Advantage A10 185 UV Ultrapure Water System (18.2 MΩ; Molsheim, France). Cu–Tria was synthesized according Kowol *et al.*,^34^ COTI-2 and Cu–COTI following procedures from Bormio Nunes *et al.*^31^

### Preparation of drug-loaded liposomes

#### Liposomal formulation of Cu–COTI (l-Cu–COTI)

The liposomes of Cu–COTI were prepared by *in situ* complexation of COTI-2 (2 mg) with CuSO_4_ · 5 H_2_O (1.36 mg) in 1 mL of CHCl_3_/MeOH (4 : 1, v/v). Afterwards, 20 mg DSPC, 6.5 mg DSPE–mPEG(2000), 7.2 mg cholesterol were dissolved in 4 mL of a mixture of CHCl_3_/MeOH (4 : 1, v/v), transferred to the complex solution and stirred for 1.5 h at 65 °C. The solvent was then carefully removed under reduced pressure to form a thin, yellowish film, which was dried *in vacuo* overnight. Afterwards, the film was rehydrated with 2 mL of a 0.3 M (NH_4_)_2_SO_4_ solution and 15 glass beads were added. The mixture was rotated on a rotary evaporator for 1.5 h at 65 °C. Subsequently, the suspension was decanted and refilled to 2 mL with 0.3 M (NH_4_)_2_SO_4_ solution. To homogenize the suspension by ultra-sonication, the Bandelin ultrasonic homogenizer HD 3100 with a maximum amplitude of 20% for 3 min, 5 min, 7 min, 5 min and 12 min (each with few seconds break) was used. Non-encapsulated drug was removed from the loaded liposomes by SEC. This was performed starting with the swelling of Sephadex G50 Fine (3.0–3.5 g per batch of liposomes) in PBS (0.01 M, pH 7.4) for one hour at 90 °C. After cooling down to RT, the resulting slurry was poured into a glass column (used for conventional column chromatography) and the stationary phase was tightly packed under pressure. Afterwards, the liposomal solution was put on the column, resulting in l-Cu–COTI (and removal of free drug). The liposomes were stored at 4 °C until further usage.

#### Liposomal formulation of Cu–Tria (l-Cu–Tria)

Liposomal formulations of Cu–Tria were prepared using the remote-loading approach.^29^ 20 mg DSPC, 6.5 mg DSPE–mPEG(2000) and 7.2 mg cholesterol were dissolved in 5 mL CHCl_3_/MeOH (4 : 1, v/v) and the resulting solution was stirred for 1.5 h at 65 °C. The solvent was carefully removed under reduced pressure to form a thin, opaque film, which was dried *in vacuo* overnight. Afterwards, the film was rehydrated with 2 mL of a 0.3 M (NH_4_)_2_SO_4_ solution and 15 glass beads were added. The mixture was rotated on a rotary evaporator for 1.5 h at 65 °C. Subsequently, the suspension was decanted and refilled to 2 mL with 0.3 M (NH_4_)_2_SO_4_ solution. To homogenize the suspension by ultra-sonication, the Bandelin ultrasonic homogenizer HD 3100 with a maximum amplitude of 20% for 3 min, 5 min, 7 min, 5 min and 12 min (each with few seconds break) was used. Excess ammonium sulfate was removed by SEC. This was performed starting with the swelling of Sephadex G50 Fine (3.0–3.5 g per batch of liposomes) in PBS (0.01 M, pH 7.4) for one hour at 90 °C. After cooling down to RT, the resulting slurry was poured into a glass column (used for conventional column chromatography) and the stationary phase was tightly packed under pressure. Afterwards, the liposomal solution was put on the column, resulting in liposomes with an extraliposomal pH of 7.4. Cu–Tria (1.2 mg) was doused with the purified liposomes (around 3.5 mL) and stirred for further 1.5 h at 65 °C. To remove non-encapsulated drug, another SEC was performed (same technique and conditions as before). About half of the aqueous solution was evaporated at 40 °C on a rotary evaporator to increase the concentration. The liposomes were stored at 4 °C until further usage.

### Particles size and surface charge (zeta potential)

The PDI and particle sizes were determined by DLS with a Malvern ZetaSizer Nano ZS (Malvern Instruments Ltd, Malvern, UK) equipped with a 4 mW He–Ne, 632.8 nm laser beam at 25 °C and at a scattering angle of 173°. Prior to the particle size measurement, the liposomes and nanoparticles were diluted (1 : 9 v/v) with PBS and measured in disposable cuvettes (UV-cuvette micro, Brand GmbH + Co KG, Germany). The zeta-potential was determined in disposable folded capillary cells using the same instrument.

### TEM measurements

The liposomes were analyzed by negative stain electron microscopy using a Carl Zeiss Libra 120 electron microscope. The liposomal formulation was diluted (1 : 10 v/v, with Millipore water), 10 μL of the resulting solution were pipetted on a carbon-coated G240-mesh Nylon grid (Agar Scientific) and the grid was allowed to dry overnight. At the next day, the grid was placed on a drop of Uranyless (seated on a piece of parafilm tape) for 1 min. Excess solvent was removed with a filter paper and the grid was dried for 10 min. The grid was then again placed on a fresh drop of Uranyless and after 1 min the excess was drawn off with a filter paper. After drying for 3 h, the grid was analyzed with the electron microscope.

### Determination of encapsulated drug amount

Aliquots of 50 μL of the liposome probes were dried under reduced pressure by rotary evaporation and then *in vacuo* for 10 min. The resulting film was dissolved in methanol and sonicated in an ultrasonic bath for 2 min. The amount of encapsulated drug was determined by UV-Vis spectroscopy on an Agilent 8453 UV-Vis spectrophotometer (Agilent Technologies, Germany) using 10 mm path length quartz cuvettes. To examine the concentration of the samples a calibration curve of the drug was measured.

### Drug release studies

For examination of the drug release, the dialysis bag diffusion technique was used. Freshly prepared l-Cu–Tria was filled into a dialysis membrane (average flat width of 10 mm and molecular weight cut off of 14 kDa; Sigma Aldrich, Austria). The membrane was sealed and immersed into 25 mL PBS (pH = 7.4) at 37 ± 1 °C with continuous stirring at 200 rpm. To examine the amount of drug that diffused through the dialysis membrane, 1 mL-samples were withdrawn from the solution at fixed time intervals (0 min, 1 h, 3 h, 6 h, 24 h and 48 h) and replaced by 1 mL of fresh PBS. The drug concentration was measured by UV-Vis spectroscopy on an Agilent 8453 UV-Vis spectrophotometer (Agilent Technologies, Germany). As a positive control, a stock solution of the free drug Cu–Tria in water was diluted with PBS to the concentration of the drug in the liposomes. This solution was also placed into the dialysis bag and the drug content in the outer compartment was determined as described above. All experiments were performed in duplicates.

### Biological investigations

#### Cell culture

The following human cell lines were used: the colon carcinoma cell lines SW480 and HCT-116 (both obtained from American Type Culture Collection, Manassas, VA). SW480 cells were cultured in minimal essential medium and HCT-116 cell lines in McCoy's 5a medium supplemented with 2 mM glutamine (from Sigma-Aldrich, MO, US) containing 10% fetal bovine serum and kept at 37 °C and 5% CO_2_.

#### Cellular copper levels

Cells were seeded (3 × 10^5^ per well) in 1 mL in 6-well plates and left to recover for 24 h at 37 °C and 5% CO_2_. The liposome-free compounds were dissolved in PBS (1 mM) and then further diluted in growth medium. Drug dilutions were added in 1 mL per well for 3 h in triplicates. Then, cells were washed twice with PBS, left to dry overnight and lysed with 500 μl HNO_3_ per well. After one-hour incubation, 400 μl of each well were removed and diluted with 7.6 mL H_2_O for copper measurement with ICP-MS (see below).

#### Viability assay

Cells were seeded (2 × 10^4^ cells per well) in 100 μl per well in 96-well plates and allowed to attach for 24 h at 37 °C and 5% CO_2_. The liposome-free compounds were diluted in PBS (1 mM) and then further diluted in growth medium. Drug dilutions were added in 100 μl per well, with the final concentrations depending on the compound and the cell line. After drug treatment, cells were incubated for 48 h or 72 h at 37 °C and 5% CO_2_. The proportion of viable cells was determined by 3-(4,5-dimethylthiazole-2-yl)-2,5-diphenyltetrazolium assay (MTT) following the manufacturer's recommendations (EZ4U, Biomedica, Vienna, Austria). Anticancer activity was expressed as IC_50_ values (drug concentrations inducing 50% reduction of cell survival in comparison to the control) calculated from full dose–response curves using GraphPad Prism software.

#### Clonogenic assay

For long-term drug exposure, 200 cells per well were seeded in 24-well plates and allowed to recover for 24 h. Then, the cells were exposed to the compounds for 10 days. The cells were fixed with methanol (−20 °C, 20 min) and after washing with PBS stained with crystal violet (1 h, 100 μg ml^−1^, Sigma-Aldrich, Austria). The washed and dried plates were then measured for fluorescence (with 633 nm excitation and 610/30 nm BP emission filter) with the imager Typhoon Trio (GE Healthcare Life Sciences). The sum of fluorescence intensities per well (integrated density) was measured with ImageJ and, after background subtraction, normalized to untreated cells.

#### Immunofluorescence

Cells (8 × 10^4^ mL^−1^) were seeded in 50 μL on 10-well PTFE printed spot slides (Science Services, Austria). After 24 h recovery, cells were treated with indicated drug concentrations and fixed with 4% paraformaldehyde for 10 min at room temperature and (after washing with PBS) blocked and permeabilized with a solution containing 5% bovine serum albumin (BSA), 0.3% Triton-X-100 in PBS for 1 h. The primary antibody pH2A.X (#2577, Cell Signaling Technology, Austria) was added 1 : 200 in a solution containing 1% BSA and 0.3% Triton-X-100 in PBS overnight at 4 °C. After washing with PBS, the cells were incubated with anti-rabbit secondary antibody conjugated to AlexaFluor488 (#A-11008, Thermo Fisher, 1 : 500 in 1% BSA and 0.3% Triton-X-100 in PBS) for 1 h. The cells were again washed and counterstained with 4′,6-diamidine-2′-phenylindole dihydrochloride (DAPI; 1 μg mL^−1^) in PBS for 10 min. The dyes were removed, and the cells mounted in Vectashield mounting medium (Vector Laboratories, CA, USA) with a coverslip. Images were taken with a Zeiss LSM 700 Olympus (Carl Zeiss AG, Oberkochen, Germany) confocal microscope and pH2A.X fluorescence intensities per nucleus were measured using ImageJ.

#### Protein isolation and western blot

Cells were seeded 2 × 10^5^ per well or 2 × 10^4^ per well in 6-well plates. After 24 h recovery, cells were treated for 24 h or 7 days, respectively, with the indicated concentrations of the compounds. Cells were scratched into the medium and after washing with PBS, lysed in lysis buffer (50 mM Tris, 300 mM NaCl, 0.5% Triton-X-100) containing a phosphorylation inhibitor cocktail (Complete and Phospho-Stop, Roche, Austria) for 45 min on ice. After 5 min ultrasound bath, lysates were centrifuged for 15 min at 14 000 rpm at 4 °C. Protein concentration of supernatant was quantified using Micro BCA Protein Assay (Pierce, Thermo Fisher, Austria). 15 μg of proteins were separated by SDS-PAGE (10% gels), and transferred onto a polyvinylidene difluoride membrane for western blotting as described previously.^51^ The following primary antibodies were used: anti-pH2AX monoclonal rabbit (Cell Signaling, #2577) and anti-β-actin monoclonal mouse (Sigma Aldrich, #A5441). Secondary, horseradish peroxidase-labeled antibodies were anti-rabbit monoclonal mouse (sc-2357, Santa Cruz Biotechnology, Austria) and anti-mouse polyclonal goat (Merck, #A0168) were used in working dilutions of 1 : 10 000.

#### Animals

Eight-to twelve-week-old BALB/c mice were purchased from Janvier (France). The animals were kept in a pathogen-free environment and every procedure was done in a laminar airflow cabinet. Experiments were done according to the regulations of the Ethics Committee for the Care and Use of Laboratory Animals at the Medical University Vienna (proposal number BMWF-66.009/0157-V/3b/2019), the U.S. Public Health Service Policy on Human Care and Use of Laboratory Animals as well as the United Kingdom Coordinating Committee on Cancer Prevention Research's Guidelines for the Welfare of Animals in Experimental Neoplasia. To ensure animal welfare throughout the experiment, the body weight of the mice was assessed once a day. At weight loss exceeding 10% (in less than two days), animals were sacrificed by cervical dislocation.

#### Pharmacokinetic experiments

In order to reduce the number of used animals (following the “3R” guidelines), rotation design was used for this experiments. In more detail, as a first step, blood was sampled from six untreated female BALB/c mice *via* the facial vein. Seven days later, the animals received their first i.v. treatment (three animals per group) with either 1.75 mg kg^−1^ Cu–Tria (in 5% glucose in physiological saline) or l-Cu–Tria (in PBS, 100 μl/20 g mouse). In each treatment group, blood was again drawn *via* facial vein after 3, 24 or 48 h from one mouse per group. After a wash-out/recovery period of seven days, the process was repeated at different collection time points for each mouse. This was then performed a third time. Thus, finally the 3, 24 and 48 h time points were collected for each mouse. After blood coagulation, serum was separated from the blood pellet by two centrifugation steps (3000*g*, 10 min) and stored at −20 °C for ICP-MS analysis.

#### ICP-MS measurements

The copper levels in cell lysates and serum were determined by ICP-MS. The cell lysates were prepared as described above (see Materials and Methods: Cellular copper levels). Preparation of the serum samples was performed *via* digestion, parameters are stated below (Table 2).

**Table 2:** Experimental parameters of sample preparation for the ICP-MS measurements

Sample preparation	
Type	Open vessel graphite digestion
Solution	2 mL HNO_3_ (20%) + 0.1 mL H_2_O_2_ (30%)
Vial material	PFA
Vial size	25 mL
Weighing	Approx. 10–30 mg

For measurements the ICP-MS Agilent 7800® (Agilent Technologies, Tokyo, Japan) was equipped with an Agilent SPS 4 autosampler (Agilent Technologies, Tokyo, Japan) and a MicroMist nebulizer at a sample uptake rate of approx. 0.2 mL min^−1^. The Agilent MassHunter® software package (Workstation Software, version C.01.04, Build 544.17, Patch 3, 2018) was used for data processing. The experimental parameters for ICP-MS are summarized in the Table 3. The instrument was tuned on a daily basis to achieve maximum sensitivity. The instrumental LOQ (LOQ = blank average + 10 × stdev) was calculated by measuring a clean blank 5 times. All Cu-values were blank corrected.

**Table 3:** Experimental parameters of the ICP-MS measurements

ICP-MS Agilent 7800	
RF power [W]	1550
Cone material	Nickel
Carrier gas [L min^−1^]	1.07
Plasma gas [L min^−1^]	15
Monitored isotopes	^115^In, ^63^Cu, ^65^Cu
Mode	No gas, O_2_
Integration time [s]	0.1
Number of replicates	10
Number of sweeps	100

#### Investigation of methemoglobin formation

Fresh solutions of HbO_2_ were prepared by dissolving human hemoglobin (20 mg) in 1 mL of PBS and reducing the metalloprotein by incubation with an excess of sodium dithionite (∼4 mg) for 1.5 h at room temperature. Afterwards, the resulting solution was purified by gel filtration (Sephadex G-25 Fine and PBS as eluent; for details see preparation of drug-loaded liposomes). The final concentration of HbO_2_ was determined by UV-Vis spectroscopy from the extinction coefficient of the protein at 542 nm.^48^ Stock solutions of Cu–Tria (5 mM) and Fe(NO_3_)_3_ (50 mM) were prepared in MeOH. l-Cu–Tria and Cu–Tria were then incubated with Fe(NO_3_)_3_ in a 2 : 1 molar ratio for 10 min at room temperature and diluted with PBS. For the kinetic studies, the incubated solutions (final concentration of compounds: 50 μM) and HbO_2_ (final concentration: 5 μM total heme) in PBS were measured over a period of 22 min using a PerkinElmer UV-Vis spectrometer Lambda 35 over 15 cycles (300–650 nm; scan-speed 480 nm min^−1^). As negative control, all compounds were additionally measured without prior incubation with Fe(NO_3_)_3_ (final concentration of compounds: 50 μM and HbO_2_: 5 μM total heme) and no changes in the UV-Vis spectra could be observed (data not shown). For quantification the concentrations of HbO_2_ and metHb were determined at 577 nm and 630 nm after subtraction of the controls (HbO_2_ or the compound alone at 630 nm) from the extinction coefficient.^48^

## Author contributions

MM performed the chemical synthesis, encapsulation and characterization of the liposomal formulations as well as the methemoglobin formation assay. SH conducted and analyzed the biological investigations, including MTT and long-term viability measurements, immunofluorescence staining, western blot as well as the sample preparation for ICP-MS measurements. MM, SH, CRK and PH contributed to the writing of the manuscript as well as the study design and conception. BKK helped with the study design and critically revised the manuscript.

## Conflicts of interest

The authors declare no competing interest.

## Supplementary Material

DT-050-D1DT02763H-s001
