# Supplementary material for: Liposomal formulations of anticancer copper(ii) thiosemicarbazone complexes
Source: Dalton Trans. 2021 Oct 7;50(44):16053–66. doi: 10.1039/d1dt02763h (PMC8594434; doi:10.1039/d1dt02763h)
Supplement: DT-050-D1DT02763H-s001 [file DT-050-D1DT02763H-s001.pdf]

Electronic Supplementary Material (ESI) for Dalton Transactions.
This journal is © The Royal Society of Chemistry 2021

Supporting Information

Liposomal formulations of anticancer copper(II) thiosemicarbazone complexes

Marlene Mathuber[a], Sonja Hager[b,c], Bernhard K. Keppler[a,c], Petra Heffeter[b,c] and Christian R. Kowol[a,c,]*

[a] Institute of Inorganic Chemistry, Faculty of Chemistry, University of Vienna, Waehringer Straße 42, 1090 Vienna, Austria

[b] Institute of Cancer Research and Comprehensive Cancer Center, Medical University of Vienna, Borschkegasse 8A, 1090 Vienna, Austria

[c] Research Cluster ''Translational Cancer Therapy Research'', University of Vienna and Medical University of Vienna, 1090 Vienna, Austria

E-mail address: christian.kowol@univie.ac.at

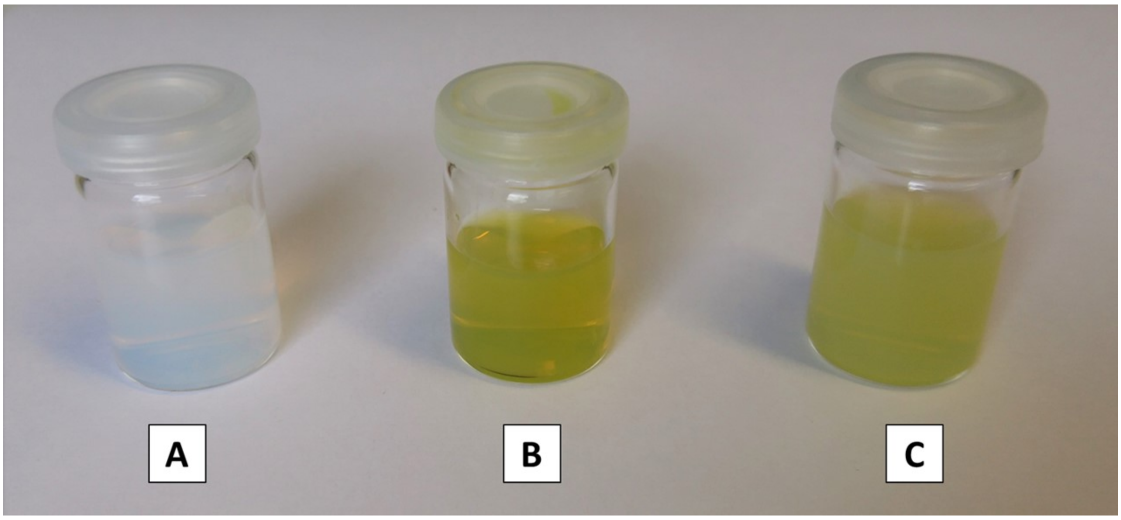

Figure S1: Pictures of (A) unloaded liposomes, (B) **L-Cu-Tria** and (C) **L-Cu-COTI**.

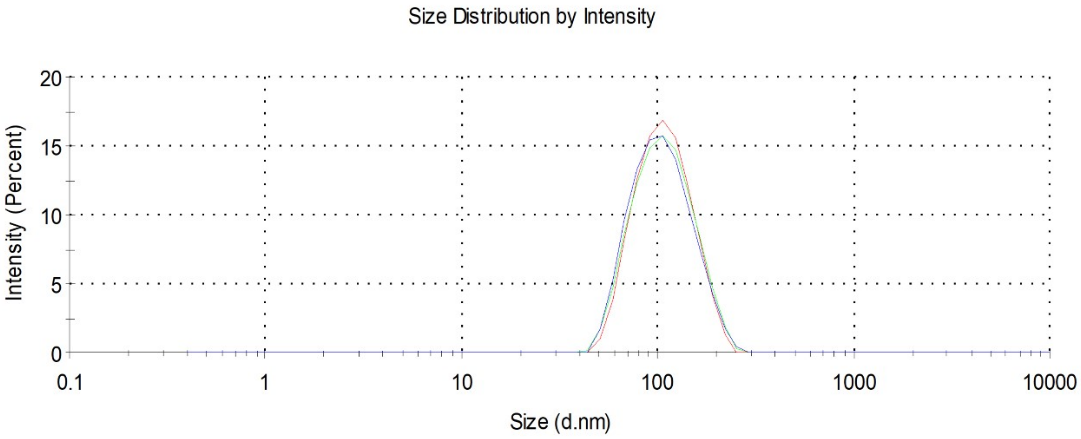

Figure S2: Size distribution of **L-Cu-COTI** (by intensity) measured by dynamic light scattering (each line represent measurements in triplicate).

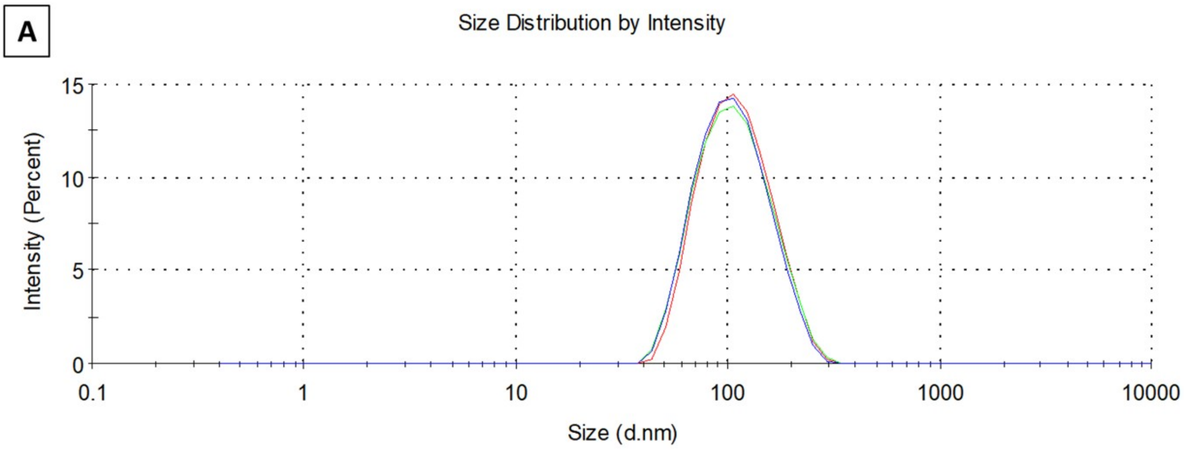

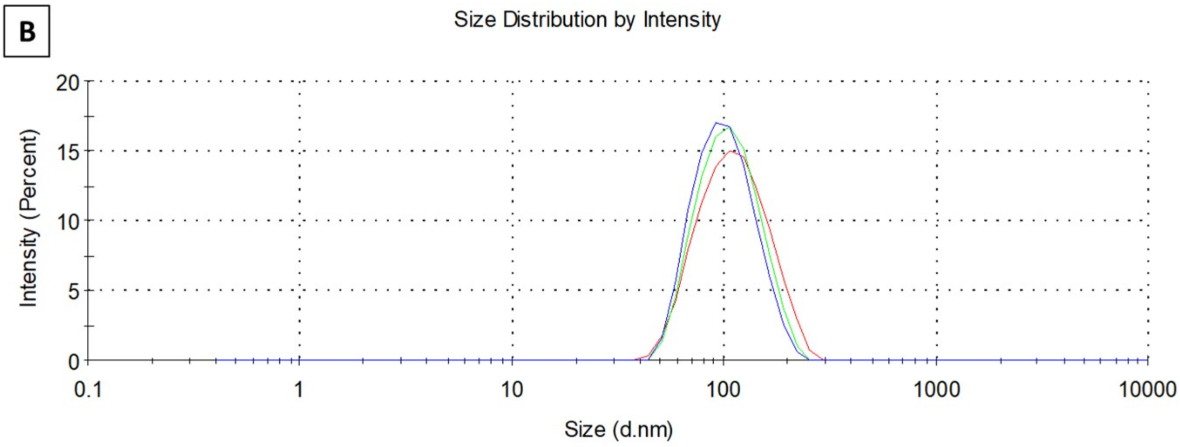

Figure S3: Size distribution of **L-Cu-Tria** (by intensity) measured by dynamic light scattering (each line represent measurements in triplicate). A) Directly after the second size exclusion chromatography B) after reducing the solution by approximately half of its volume.

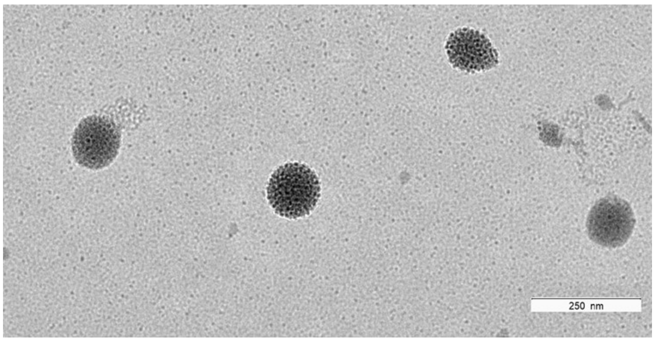

Figure S4: Transmission electron microscopy image of **L-Cu-Tria**; samples were prepared by negative staining with Uranyless.

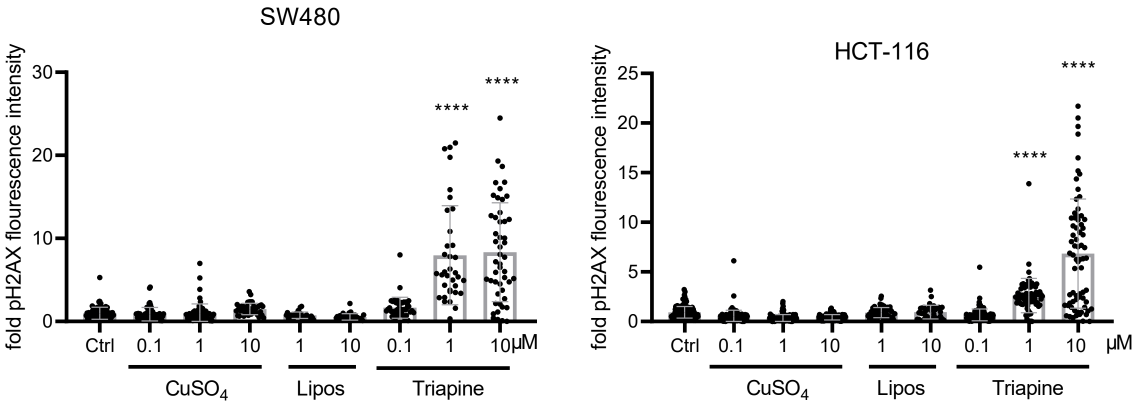

Figure S5: Quantification of immunofluorescence intensities in the nucleus of the DNA damage marker pH2AX in SW480 and HCT-116 cells treated with indicated concentrations of $CuSO_4$, empty liposomes (equivalent concentration to loaded liposomes) or Triapine for 24 h. Values given are the mean fold intensities ± SD per nucleus. Significance to control was calculated by two-way ANOVA and Dunnett's multiple comparison test (**** $p < 0.0001$).
